# Ethnopharmacological Evaluation of a Wild Ecotype of *Syzygium cumini* (L.) Skeels (Jamoya) Fruit

**DOI:** 10.1002/cbdv.71627

**Published:** 2026-08-25

**Authors:** Ritu Sharma, Umesh Pal, Jay Krishna Singh, Meenakshi Tanwar, Rajinder K. Gupta

**Affiliations:** ^1^ Department of Applied Chemistry Delhi Technological University Delhi India

**Keywords:** antidiabetic activity, jamoya, *Syzygium cumini* (L.) skeels, UHPLC‐QTOF‐MS, wild ecotype

## Abstract

The wild ecotype of Jamun (*Syzygium cumini*) is known for its inedible fruits that are not yet explored for their bioactive potential. The present study assesses the ethnopharmacological and phytochemical properties of its fruit pulp and seed extracts. GC‐MS and UHPLC‐QTOF‐MS analyses identified 44 compounds in seeds and 38 compounds in pulp. The *α*‐amylase and *α*‐glucosidase inhibitory assays revealed IC_50_ values of 0.238 and 1.65 µL/mL for pulp, and 0.05 and 4.80 µL/mL for seeds, respectively. Insulin secretion was enhanced, with pulp extract showing 297.73 U/mL and seed extract 244.45 U/mL, compared to 156.55 U/mL in the control. Glucose uptake in L6 cells was significantly increased (707 µg/mL for pulp, 697.35 µg/mL for seeds, vs. 506.48 µg/mL in the control). Cytotoxicity assays showed safe profiles, with IC_50_ values of 314 ± 0.09 µg/mL for pulp and 83.9 ± 0.1 µg/mL for seeds. Overall, the findings suggest that the wild ecotype of *Syzygium cumini* fruit, which is not consumed by humans and animals, exhibits strong antidiabetic, antioxidant, and antibacterial activities, indicating its potential for use in the Indian system of medicine.

## Graphical abstract

The phytochemical and biological activity of *Syzygium cumini* (ecotype) fruit pulp and seed extracts using GC‐MS, UHPLC‐QTOF‐MS, and antidiabetic potential.

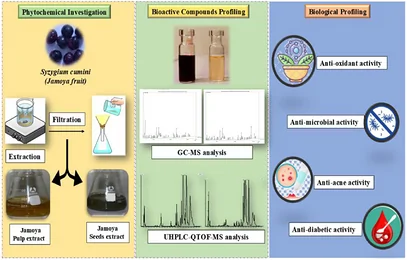

## 1 Introduction

*Syzygium cumini* (L.) Skeels (*S. cumini*, Family ‐ Myrtacaea), commonly known as Jamun, Ram jamun, Jambolan, Jambul, Jamblang, Black Plum, Blackberry, and Java Plum, is a perennial plant species found in the Indian subcontinent of Southeast Asia and China [1]. Ram jamun, which is a common variety grown in North India, is big in size, has oblong fruit, and is deep purple in color. It is commonly used in Chinese medicine to treat digestive disorders and has a high concentration of vitamins A and C. It exhibits various biological properties, including antioxidant, antidiarrheal, antimicrobial, anti‐inflammatory, potential against hypoglycemia and hyperglycaemia, cardioprotective, and hypolipidemic properties [2, 3].

**FIGURE 1:**
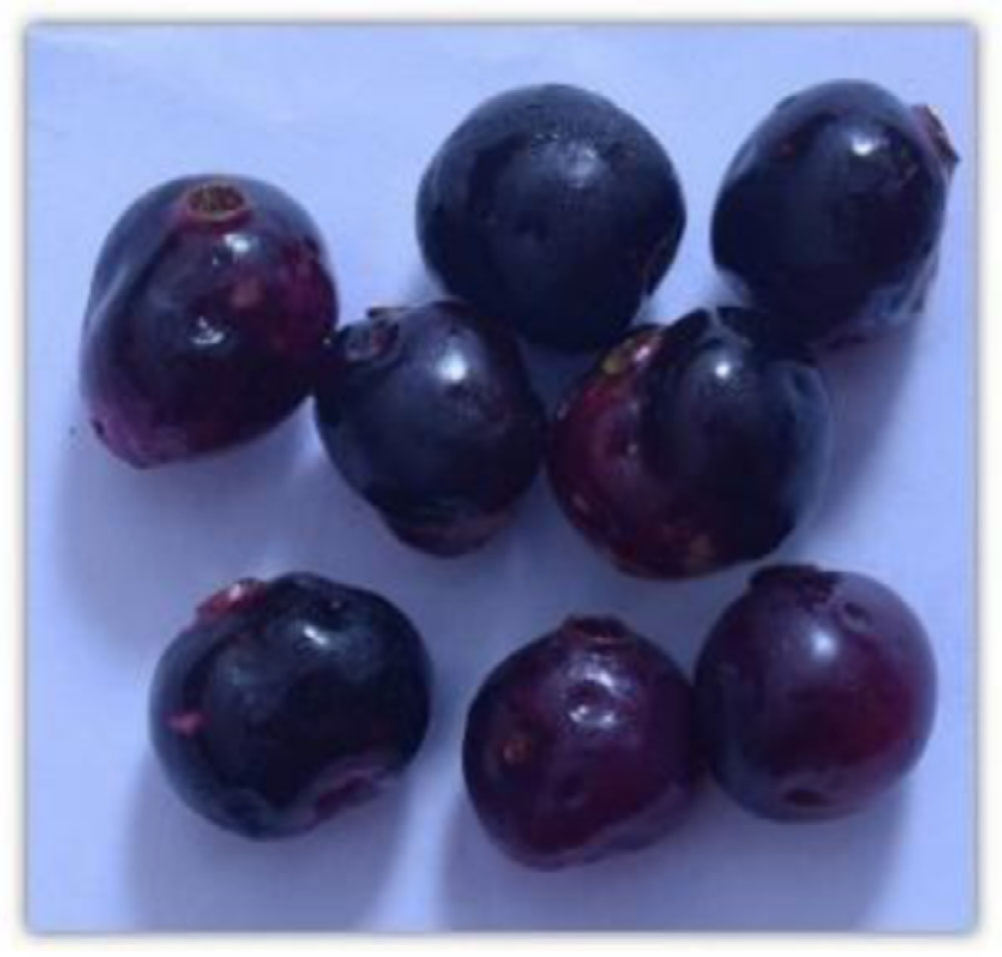
*Syzygium cumini* (L.) Skeels (Jamoya) fruit.

The wild ecotype Jamoya and the commonly consumed Ram Jamun are easily mistaken for each other because of their shared botanical family and nearly similar appearances (as shown in Figure 1). This particular ecotype is not widely consumed by humans or animals due to its unique characteristics, including its astringent taste and small size, making it a relatively underutilized source of bioactive compounds with untapped potential for health applications [4]. Exploring such unexplored/underutilized wild ecotypes aligns with the growing interest in diversifying natural resources for herbal and complementary medicines. Based on the extensive literature survey, it was found that virtually no studies discuss the antidiabetic and antiacne activity of the wild ecotype varieties of *S. cumini*. Furthermore, reports that showcase the differentiation between various ecotypes of jamun are severely lacking.

Although *Syzygium cumini* (Ram jamun, edible variety) has been investigated, the naturally occurring wild ecotype (Jamoya) has received limited scientific attention and has not been comprehensively characterized as a distinct ecotype with respect to its phytochemical composition and biological activities. As genetic diversity and ecological adaptation may influence secondary metabolite profiling, we hypothesized that the wild ecotype may possess a distinct metabolite profile and associated biological activities. Therefore, the present study aimed to comprehensively characterize its phytochemical composition and biological properties to provide a scientific basis for its potential value‐added utilization in the Indian system of medicine. Current study evaluates (a) in vitro antidiabetic activity of *S. cumini* pulp and seeds by the *α*‐glucosidase, glucose uptake assay, *α*‐amylase assay, reactive oxygen species (ROS) activity, and insulin secretion assay. (b) Antioxidant activity by the method of DPPH radical scavenging assay. (c) Antifungal and antibacterial activity by disc‐diffusion method (d) cytotoxicity activity by MTT assay. The bioactive compounds in *S. cumini* pulp and seed extracts were analyzed using UHPLC‐QTOF‐MS (Ultra High‐Performance Liquid Chromatography‐Quadrupole Time of Flight‐Mass Spectrometry) and GC‐MS (Gas Chromatography‐Mass Spectrometry) analysis. To the best of our knowledge, this is the first study on the phytochemical and biological profiling of the wild ecotype variety of *S. cumini* (L.) Skeels (Jamoya) fruits. The findings are expected to shed light on its potential as a natural antidiabetic agent for diabetes and emphasize its significance in traditional and modern healthcare systems.

## 2 Experimental Section

### 2.1 Plant Material Collection and Authentication

*S. cumini* fruits were collected in the month of June from the trees on campus, Delhi Technological University, Delhi, India. The fruit samples were verified by Dr. Sunita Garg, former emeritus and chief scientist at CSIR‐NIScPR, Raw Materials Herbarium and Museum (RHMD), Delhi, India, and the voucher sample (voucher specimen number: NIScPR/RHMD/Consult/2023/4618‐19) was placed at the RHMD, Delhi.

### 2.2 Extraction of Plant Material

Fifty grams of crushed seeds and pulp were extracted individually with methanol by following the previously described method. After the complete extraction, the samples were filtered using a muslin cloth. The solvent was evaporated using a rotary evaporator. The concentrated extracts were stored at 4°C until further use [5].

### 2.3 Phytochemical Analysis

Methanolic extracts of *S. cumini* seeds and pulp were analyzed qualitatively for tannins, terpenoids, saponins, glycosides, proteins, amino acids, phenolic compounds, and coumarins [6].

### 2.4 GC‐MS Analysis

The quantitative analysis of volatile compounds in the methanol extracts of *S. cumini* seeds and pulp was determined using Shimadzu, GCMS‐QP2010 instrument, as per the previously described method [7]. The details of all the experimental methods performed have been provided in Supplementary Information.

### 2.5 UHPLC‐QTOF‐MS Analysis

The qualitative determination of secondary metabolites was done using the UHPLC‐QTOF‐MS, following the protocol previously described [8]. The mass spectroscopic analysis was carried out only in positive mode (ESI+). The conditions of the ESI source were as follows: source temperature, 120°C; cone gas flow, 50 L/h; desolvation gas flow, 950 L/h; capillary voltage, 3.22 keV. The UHPLC system was assembled with an AD autosampler, and the column used was a Waters nanoAcquity HSS paired with a quadrupole time‐of‐flight mass spectrometer (QTOF‐MS). An Accucore C18 column measuring 100 mm by 3 mm made up the analytical column. Subsequently, a binary mobile phase comprising various components labeled as A (water containing 0.1% formic acid) and B (1% formic acid in acetonitrile) was used to elute 5 µL of methanol extracts (1 mg/mL) into the column. A linear gradient at a rate of 0.2 mL/min was determined for a 25 min program. In these conditions, the scanning time was roughly 1 s. The tentative components in both samples were identified using ChemSpider software.

### 2.6 Antioxidant Activity (DPPH Radical Scavenging Activity)

The free radical scavenging activity of both methanolic extracts was evaluated following the previously reported method with few modifications [9]. The radical scavenging activity was determined using the following Equation 1:

(1) $$\begin{array}{ccc} & & \%\;\mathrm{DPPH}\;\mathrm{Scavenging}\;\mathrm{activity}\;\\ & & \;=1-\frac{\mathrm{Absorbance}\;\mathrm{of}\;\mathrm{control}-\mathrm{Absorbance}\;\mathrm{of}\;\mathrm{sample}}{\mathrm{Absorbance}\;\mathrm{of}\;\mathrm{control}}\times 100 \end{array}$$

### 2.7 Antifungal Activity (Agar Well Diffusion Method and MIC Determination)

The agar well diffusion method was used to evaluate the antifungal activity of both the methanolic extracts against *Candida albicans* [10]. Amphotericin B was taken as a positive control. The minimum inhibitory concentration (MIC) of both the methanolic extracts of *S. cumini* against selected bacterial strains was determined using the broth microdilution method following the standard protocols [11]. The microbial cultures were obtained from the Microbial Type Culture Collection (MTCC), Chandigarh, India.

### 2.8 Antibacterial Activity (Disc Diffusion Method and MIC Determination)

The disc diffusion method was used to determine the antibacterial activity of the methanolic extracts of *S. cumini* fruits against *E. coli*, following the previously described method [12]. Ciprofloxacin was taken as a positive control. The MIC values were determined for both the methanolic extracts of *S. cumini* against selected bacterial strains using the broth microdilution method following the standard protocols [11].

### 2.9 Antiacne Activity Assay (Disc Diffusion Method and MIC Determination)

The disc diffusion method was used to determine the antiacne activity of the methanolic extracts of *S. cumini* fruits against *Propionibacterium acnes* (*p. acnes)*, following the method described by Hudzicki [13]. Ciprofloxacin was taken as a positive control. The MIC values were also determined for both the methanolic extracts of *S. cumini* against selected bacterial strains using the broth microdilution method following the standard protocols [11].

### 2.10 In Vitro Biological Activity (Antidiabetic activities)

#### 2.10.1 α‐Amylase Inhibition Assay

*α*‐Amylase inhibitory activity of *S. cumini* fruit extracts was done by following the procedure followed by Telagari, with a few modifications [14]. The *α*‐amylase inhibitory activity was calculated using the following Equation 2:

(2) $$\begin{array}{ccc} & & \%\;\mathrm{Inhibition}\\ & & \;=\frac{\mathrm{Absorbance}\;\mathrm{of}\;\mathrm{control}-\mathrm{Absorbance}\;\mathrm{of}\;\mathrm{sample}}{\mathrm{Absorbance}\;\mathrm{of}\;\mathrm{control}}\;\times 100 \end{array}$$

#### 2.10.2 *α*‐Glucosidase Inhibitory Assay

The previously reported method was followed to assess the *α*‐glucosidase inhibitory activity of both fruits’ extracts with slight modifications [14]. The *α*‐glucosidase inhibitory activity was calculated using the following Equation 3:

(3) $$\mathrm{Inhibitory}\;\mathrm{activity}\;\%=1-\frac{\mathrm{Absorbance}\;\mathrm{of}\;\mathrm{sample}}{\mathrm{Absorbance}\;\mathrm{of}\;\mathrm{control}}\times 100$$

#### 2.10.3 ROS Activity Measurement

2'‐7'‐Dichlorodihydrofluorescein diacetate (DCFH‐DA) probe was utilized to produce ROS. The method of Al‐Oqail was followed to assess ROS measurement of both fruit extracts on the L6 cell line, with slight modifications [15].

#### 2.10.4 Insulin Secretion Assay

Insulin was measured using an immunoassay kit from DiaMetra (catalog no: DKO076). The insulin secretion assay was done using the modified protocol previously described by Kaur et al. [16].

#### 2.10.5 Glucose Uptake Assay

Extracts of *S. cumini* fruits were used to determine the glucose uptake assay on the L6 cell line (L6‐ Rat skeletal muscle myoblast cell line, RRID‐ CVCL_0477, obtained from NCCS, Pune, India), following the method described by Vishwanath, with slight modifications [17]. 2‐DG6P (0.1 U/mL) standard of insulin was employed as the control positive, and 1 mg of metformin as the control negative.

#### 2.10.6 Cytotoxicity Assay

The cytotoxicity of both fruit extracts was evaluated on the L6 cell line (L6‐ Rat skeletal muscle myoblast cell line, RRID‐ CVCL_0477, obtained from NCCS, Pune, India) by MTT assay, following the previously described method [18].

#### 2.10.7 Statistical Analysis

All the data were obtained from three independent experiments in triplicate (*n* = 3) and were expressed as means ± standard deviation (*p* < 0.05). The t‐test was used to determine the statistical significance of the differences between the groups. The IC_50_ value was determined from a dose‐response curve using Microsoft Excel.

## 3 Results and Discussion

### 3.1 Phytochemical Analysis

The results of phytochemical analysis revealed that saponins, carbohydrates, flavonoids, glycosides, terpenoids, and coumarins were present in the methanol extract of *S. cumini* pulp, while tannins, phlobatannin, proteins, amino acids, and phenolic compounds were found to be absent (as shown in Table 1). The methanolic extract of seeds also showed the presence of tannins, saponins, carbohydrates, terpenoids, flavonoids, glycosides, and phenolic compounds, as well as the absence of phlobatannin, proteins, and amino. The phytochemicals found in seeds and pulp extract are considered biologically active compounds, and they are found to exhibit various biological activities such as anticancer, anti‐inflammatory, antioxidant, and many more. Flavonoids show antidiabetic, anticancer, anti‐inflammatory, and anticytotoxic properties [19]. Tannins also show antidiabetic, anti‐inflammatory, antioxidant, and antimicrobial properties [20]. Saponins are responsible for anticancer and antibacterial properties [21].

**TABLE 1:** Phytochemical analysis of *Syzygium cumini* pulp and seeds in methanol extract.

Phytochemicals	Test name	Pulp extract	Seed extract
Tannins	Braymer's test	—	+
Saponins	Foam test	+	+
Phlobatannins	HCl test	—	—
Carbohydrate	Molisch's test	+	+
Flavonoids	Alkaline reagent test	+	+
Glycoside	Keller‐Killiani test	+	+
Terpenoids	Salkowski test	+	+
Proteins and Amino acids	Ninhydrin test	—	—
Phenolic Compound	Ferric chloride test	—	+

*Note*: (+) denotes the presence of phytochemicals and (–) denotes the absence of phytochemicals.

### 3.2 GC‐MS Analysis

The GC‐MS data revealed the presence of nineteen volatile compounds in the methanolic extract of *S. cumini* pulp and 32 compounds in the seed extract. The major compounds (with the highest percentage peak area) in the pulp extract were found to be 5‐[hydroxymethyl]‐2‐furaldehyde, pyranone, 2‐furoic acid, and 5‐oxy‐dimethylene‐bis(2‐furaldehyde). Similarly, the major compounds in the seed extract were found to be 5‐oxymethylfurfurole, pyranone, pyrogallol, and cycloisolongifolene, as shown in Table 2.

**TABLE 2:** Bioactive compounds identified by GC‐MS in the methanol extract of *S. cumini* pulp and seed extracts.

Compound name	RT (min)	Percentage area	MW (g mol^−1^)	MF	RRI
Pulp extract					
2‐Butenolide	3.377	0.27	84	C_4_H_4_O_2_	923
Heptanol	3.490	0.11	114	C_7_H_14_O	932
Furan‐2‐carboxylic acid	3.713	0.48	112	C_5_H_4_O_3_	949
5‐Methylfuran‐2‐al	3.957	1.33	110	C_6_H_6_O_4_	969
2,4‐Dihydroxy‐2,5dimethyl‐3(2H) furnone	4.200	1.81	144	C_6_H_8_O_4_	988
Levulinic acid	5.130	1.97	116	C_5_H_8_O_3_	1053
5‐Formylfurfural	5.597	1.20	124	C_6_H_4_O_3_	1084
2‐Furoic acid	5.680	5.08	126	C_6_H_6_O_3_	1090
n‐Nonyl acetate	6.127	0.23	186	C_11_H_22_O_2_	1119
γ‐Pyranone	6.707	12.76	144	C_6_H_8_O_4_	1158
2‐Methyl‐2H‐pyran‐3,4,5(6H)‐trione	7.237	0.61	142	C_6_H_6_O_4_	1193
1‐Methylbutyl propionate	7.470	0.48	144	C_8_H_16_O_2_	1209
5‐[Hydroxy methyl]‐2‐furaldehyde	8.120	60.61	126	C_6_H_6_O_3_	1254
1‐[(Isopentyloxy)methoxy]‐3‐methylbutane	10.323	0.70	188	C_11_H_24_O_2_	1412
Hexose	11.873	2.76	180	C_6_H_12_O_6_	1534
(Z)‐9‐Octadecenoic acid	16.603	0.19	282	C_18_H_34_O_2_	1963
5‐5’‐Oxy‐dimethylene‐bis(2‐furaldehyde)	16.957	4.33	234	C_12_H_10_O_5_	1998
3,4‐Furandimethanol	18.313	0.40	128	C_6_H_8_O_3_	2143

Seeds extract					
2‐Oxepanone	3.363	0.44	114	C_6_H_10_O_2_	922
5‐Methyl furfural	3.960	0.59	110	C_6_H_6_O_2_	969
2,4‐Dihydroxy‐2,5‐dimethyl‐3(2H)‐furanone	4.197	1.51	144	C_6_H_8_O_4_	988
4‐Hydroxy‐3‐hexanone	4.677	0.19	116	C_6_H_12_O_2_	1018
2,5‐Dimethyl‐3(2H) furanone	5.277	0.09	128	C_6_H_8_O3	1062
2‐Methyl‐3‐hydroxy‐4‐pyrone	5.943	0.14	126	C_6_H_6_O_3_	1107
Nonyl acetate	6.123	0.17	186	C_11_H_22_O_2_	1119
5‐(Hydroxymethyl)furan‐2‐carbaldehyde	6.420	0.14	126	C_6_H_6_O_3_	1139
γ‐Pyranone	6.663	9.84	144	C_6_H_8_O_4_	1155
5‐Hydroxy‐2‐(hydroxymethyl) pyran‐4‐one	7.217	0.47	142	C_6_H_6_O_4_	1192
Tetrahydro‐5‐methyl‐2‐furanmethanol	7.443	0.61	116	C_6_H_12_O_2_	1207
5‐Oxymethylfurfurole	8.007	45.78	126	C_6_H_6_O_3_	1246
(4S)‐1‐Methyl‐4‐(6‐methylhepta‐1,5‐dien‐2‐yl) cyclohexene	10.123	0.60	204	C_15_H_24_	1397
Pyrogallol	10.250	4.24	126	C_6_H_6_O_3_	1406
(‐)‐Isoledene	10.887	0.53	204	C_15_H_24_	1456
1,6‐Dimethyl‐4‐propan‐2‐yl‐1,2,3,4‐tetrahydronaphthalene	11.117	0.50	202	C_15_H_22_	1473
2‐Isopropenyl‐4a,8‐dimethyl‐1,2,3,4,4a,5,6,7‐octahydronaphthalene	11.253	0.23	204	C_15_H_24_	1484
α‐Amorphene	11.317	0.23	204	C_15_H_24_	1489
α‐Cubeben	11.373	0.28	204	C_15_H_24_	1493
β‐Selinene	11.490	0.24	204	C_15_H_24_	1502
(‐)‐α‐Gurjunene	11.637	3.00	204	C_15_H_24_	1514
Hexose	11.773	1.47	180	C_6_H_12_O_6_	1525
Cycloisolongifolene	12.520	3.12	202	C_15_H_22_	1587
β‐Vetivenene	12.563	2.20	202	C_15_H_22_	1590
1,1,4,7‐Tetramethyldecahydro‐1H‐cyclopropa[E]azulen‐4‐ol	12.720	1.07	222	C_15_H_26_O	1603
1,1,7,7a‐Tetramethyl‐1a,2,6,7,7a,7b‐hexahydro‐1H‐cyclopropa[a]naphthalene	13.083	1.01	202	C_15_H_22_	1635
Zizanal	14.663	0.13	218	C_15_H_22_O	1775
1‐[2‐(2,2,6‐Trimethylbicyclo[4.1.0]hept‐1‐yl)ethyl]vinyl acetate	15.267	2.22	250	C_16_H_26_O_2_	1832
Hexadecanoic acid	16.590	0.24	256	C_16_H_32_O_2_	1961
Cis‐linoleic acid methyl ester	17.847	0.09	294	C_19_H_34_O_2_	2092
(E)‐Phytol	18.007	0.17	296	C_20_H_40_O	2109
β‐Dihydrofucosterol	28.597	0.56	414	C_29_H_50_O	2959

RT, retention time; MF, molecular formula; MW, molecular weight; RRI, relative retention index.

Based on the data, it appears that various biologically relevant phytochemical compounds are present, which may contribute to the pharmacological properties of the S. *cumini* extract. Pyragallol, a phenolic compound, exhibits strong antifungal, antioxidant, anticancer, and antimicrobial properties [22]. (E)‐Phytol has been extensively reported to exhibit activities including antimicrobial, antioxidant, antidiabetic, and anti‐inflammatory [23]. It has been reported that 5‐[hydroxymethyl]‐2‐furaldehyde has antioxidant activity, while pyranone shows antidiabetic, antifungal, and antileishmanial activities [24, 25]. Palmitic acid has antimicrobial and antioxidant properties [23]. Linoleic acid methyl ester, a derivative of linoleic acid, may possess antiacne and antiarthritic activities [23]. Dihydrofucosterol, a phytosterol, shows antioxidant and antidiabetic effects.

### 3.3 UHPLC‐QTOF‐ MS Analysis

UHPLC‐QTOF‐MS analysis was carried out to tentatively determine the secondary metabolites present in the methanol extract of *S. cumini* seeds and pulp (Table 3). The data revealed the presence of 12 tentative secondary metabolites, including gibberellin A44 diacid, gypsogenic acid, (‐)‐pipecolic acid, and gypsogenin in the seed extract. *S. cumini* pulp extract showed the presence of 19 tentative secondary metabolites, including geraniol acetate, gypsogeninic acid, linoleic acid, geroquinol, and costundide. Among all the tentative metabolites, curcumin, a well‐known polyphenol, is reported to possess potent antioxidant, antimicrobial, anticancer, antidiabetic, and anti‐inflammatory activities [26, 27]. Leucocyanidin, a flavonoid, exhibits antioxidant activity and may possess antidiabetic activity [28, 29]. Geraniol shows various biochemical and pharmacological properties. It is one of the most important molecules in the flavor and fragrance industries, exhibiting insecticidal, repellent, antimicrobial, and antitumor properties [30]. Moreover, linoleic acid has been shown to possess antimicrobial and antiacne properties, while geraniol acetate, and gypsogenic acid have reportedly demonstrated antioxidant and antimicrobial activities [31, 32, 33, 34]. It can be inferred from the data that both extracts are a powerhouse of secondary metabolites such as phenolic compounds, lignans, alkaloids, terpenoids, and flavonoids, which are found to be active as antidiabetic, anticancer, anti‐inflammatory, antibacterial, and antioxidant [35].

**TABLE 3:** UHPLC‐QTOF‐MS of methanol extract of *Syzygium cumini* seeds and pulp extract.

Peak no.	Tentative metabolites	RT (Min)	MF	MW (gmol^−1^)	[M‐H]^+^	Error (ppm)	Compound ID
Seed extracts							
1.	4‐O‐β‐d‐glucopyranosyl‐β‐D‐glucopyranose	2.34	C_12_H_22_O_11_	319.1265	320.1343	−1.2	CSID10261
2.	6‐O‐α‐d‐Galactopyranosyl‐β‐D‐glucopyranose	2.34	C_12_H_22_O_11_	303.0179	304.0257	−1.6	CSID10974
3.	2‐Isopropyl‐3‐oxosuccinat	5.15	C_7_H_8_O_5_	132.9399	133.9477	4.0	CSID24785380
4.	Gibberellin A44 diacid	13.97	C_20_H_26_O_6_	361.1662	362.174	−2.5	CSID24784737
5.	Gypsogenic acid	21.71	C_30_H_44_O_5_	460.3224	461.3302	−0.2	CSID24784682
6.	8',10‐Diapocarotene‐8',10‐dial	22.12	C_17_H_20_O_2_	255.1385	256.1463	−1.5	CSID58829813
7.	(1S,5R,8R)‐1,5‐dimethyl‐8‐[(1E)‐3‐oxo‐1‐buten‐1‐yl]‐6‐oxabicyclo[3.2.1] octan‐3‐on	23.73	C_13_H_18_O_3_	183.1817	184.1895	2.6	CSID59700305
8.	(‐)‐Pipecolic acid	23.73	C_6_H_11_NO_2_	106.089279	107.097079	1.1	CSID388365
9.	Gypsogenin	24.51	C_30_H_46_O_4_	469.3318	470.3396	−1.3	CSID 83794
10.	Xanthoxic acid	24.51	C_15_H_22_O_4_	265.1439	266.1517	−4.1	CSID 4445404
11.	(1S,2R,4aS,6aR,6aS,6bR,8aR,9R,10R,11R,12aR,14bS)‐10,11‐dihydroxy‐9‐(hydroxymethyl)‐1,2,6a,6b,9,12a‐hexamethyl‐2,3,4,5,6,6a,7,8,8a,10,11,12,13,14b‐tetradecahydro‐1H‐picene‐4a‐carboxylic acid	24.51	C_30_H_48_O_5_	487.3423	488.3501	−1.6	CSID 24785685
12.	Lecithin	24.51	C_44_H_78_NO_8_P	778.5387	789.5465	−2.1	CSID24766802

Pulp extract							
13.	1‐Oxo‐1‐[(2‐oxo‐8‐heptadecanyl)oxy]‐9‐octadecanyl(9E)‐9‐octadecenoate	2.39	C_53_H_100_O_5_	793.7673	794.7751	3.8	CSID24785451
14.	(2R,3S,4S)‐Leucocyanidin	12.53	C_15_H_14_O_7_	266.9756	267.9834	1.6	CSID389677
15.	Eupatolitin‐3‐glucoside	14.59	C_23_H_24_O_13_	485.1320	486.139	−1.4	CSID8544432
16.	Gypsogeninic acid	22.75	C_30_H_46_O_5_	485.3267	486.3345	−3.2	CSID10217372
17.	(2R)‐1‐(β‐D‐Galactopyranosyloxy)‐3‐hydroxy‐2‐propanyl palmitate	23.54	C_25_H_48_O_9_	491.3220	492.3298	−4.0	CSID58829698
18.	2‐(2‐Methylencyclopropyl)‐3‐oxosuccinat	1.99	C_8_H_6_O_5_	181.0148	182.0226	1.2	CSID24785550
19.	Lambertine	2.20	C_20_H_19_NO_4_	314.1417	315.1495	−2.8	CSID9800
20.	Geraniol acetate	2.34	C_12_H_20_O_2_	195.1385	196.1463	−0.3	CSID1266019
21.	1‐Glycerophosphate	10.70	C_3_H_7_O_6_P	147.0094	148.0172	−1.4	CSID2939444
22.	Dioxindole‐3‐acetate	12.15	C_10_H_8_NO_4_	205.0380	206.0458	1.7	CSID24784782
23.	(1S,5R,8R)‐1,5‐Dimethyl‐8‐[(1E)‐3‐oxo‐1‐buten‐1‐yl]‐6‐oxabicyclo [3.2.1] octan‐3‐one	18.72	C_13_H_18_O_3_	221.1177	222.1255	3.6	CSID59700305
24.	Gypsogenate	21.71	C_30_H_44_O_5_	483.3121	484.3199	−4.4	CSID24784682
25.	(+) Gomisin‐M2	22.12	C_22_H_26_O_6_	385.1651	386.172943	−2.0	CSID10278518
26.	Geroquinol	23.73	C_16_H_22_O_2_	245.1541	246.1619	−1.6	CSID4445151
27.	Costundide	25.41	C_15_H_20_O_2_	231.1385	232.1463	4.1	CSID4444782
28.	11‐Hydroxylaurate	25.93	C_12_H_23_O_3_	214.1574	215.1652	−4.8	CSID24785390
29.	Linoleic acid	27.65	C_18_H_32_O_2_	279.2324	280.2402	−1.2	CSID4444105
30.	Harmidol	28.64	C_12_H_12_N_2_O	177.1052	178.1130	3.4	CSID11262879
31.	Curcumin	29.61	C_21_H_20_O_6_	367.1181	368.1259	1.4	CSID4445080

CSID, ChemSpider ID.

### 3.4 Antioxidant Activity (DPPH Radical Scavenging Activity)

**FIGURE 2:**
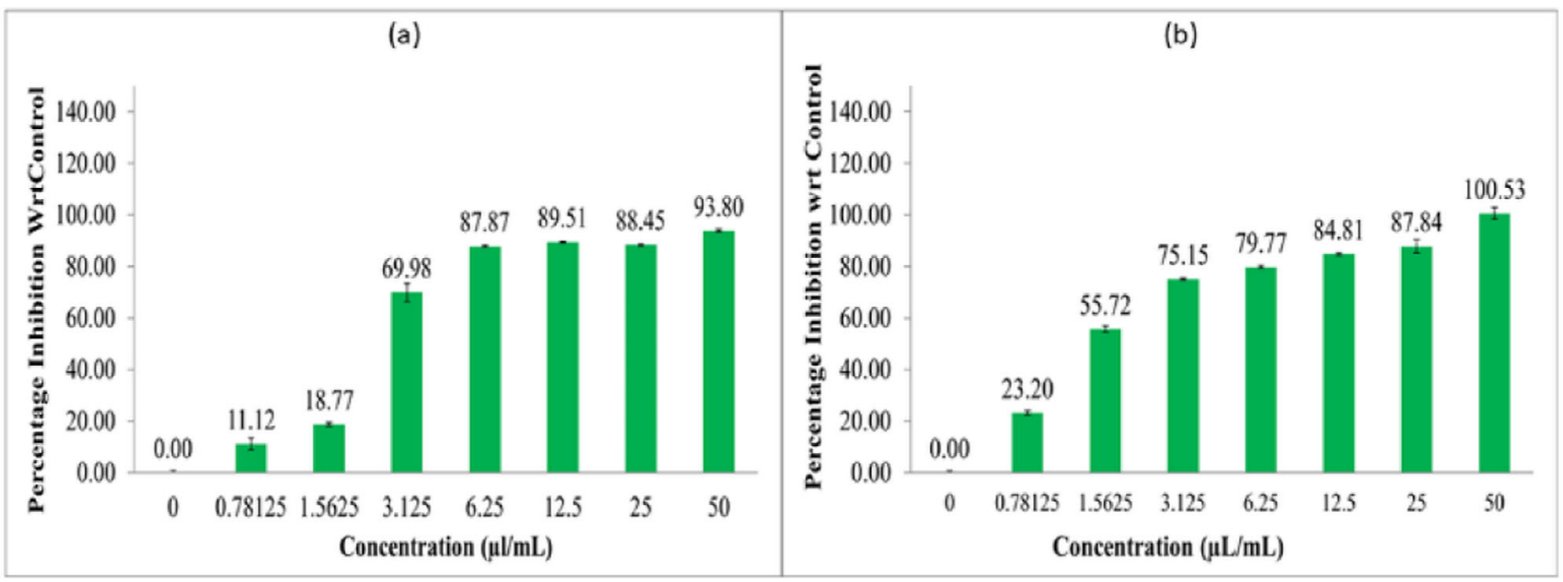
DPPH Scavenging activity of methanolic extract of *S. cumini*: (a) pulp (b) seeds.

2,2‐Diphenyl‐1‐picrylhydrazyl (DPPH^+^) is a stable free radical that has been widely used for assessing antioxidant properties. Figure 2 and Table S1 depict the radical scavenging capabilities of both extracts. The results showed that the extracts have concentration‐dependent action against free radical species. The seeds (IC_50_ = 0.1592 ± 0.068 µL/mL) and pulp (IC_50_ = 0.2469 ± 0.047 µL/mL) were found to be equivalent to (IC_50_ = 1.12 ± 0.32 µg/mL) ascorbic acid [36]. The results suggest that seed extract is more effective in scavenging free radicals than pulp extract. Thereby suggesting their potential as a natural antioxidant.

### 3.5 Antifungal Activity

#### 3.5.1 Agar Well Diffusion Method

**FIGURE 3:**
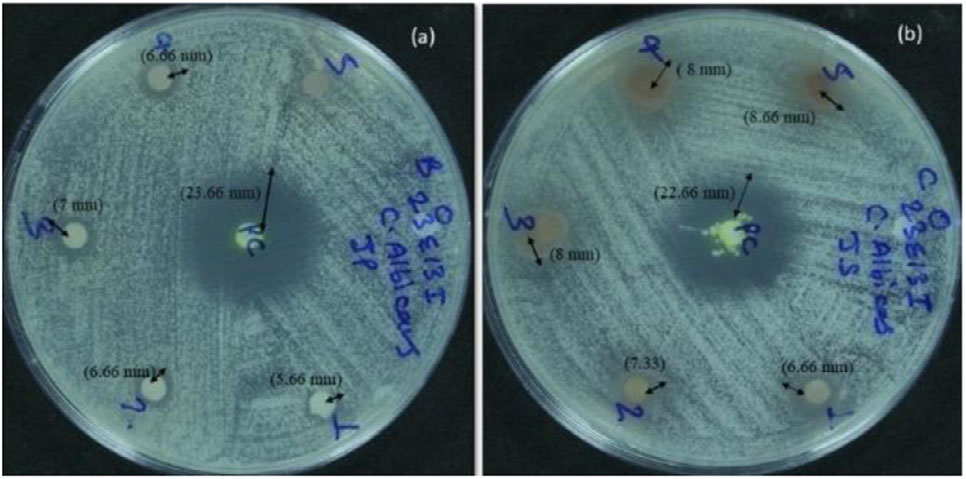
Antifungal activity of methanolic extract of *S. cumini*: (a) seeds (b) pulp.

The agar well diffusion method was used to assess the antifungal activity of *S. cumini* pulp against *C. albicans*. Figure 3a,b shows the inhibitory zones surrounding each disc in the presence of different percentage of concentrations (0%, 6.25%, 12.5%, 25%, 50%, and 100% dose) of both the methanolic extract of *S. cumini* seeds and pulp. *S. cumini* seeds (maximum zone of inhibition 8.6 mm at 6.25% dose) and *S. cumini* pulp (maximum zone of inhibition 7 mm at 25% dose) exhibited antifungal activity against the test organism *C. albicans* as compared to the positive control (maximum zone of inhibition for seeds 22.66 mm and 23.66 mm for pulp at 50 µg dose).

#### 3.5.2 MIC Determination

The antifungal activity of *S. cumini* seeds and pulp extract was evaluated by determining the MIC against *C. Albicans*. Both *S. cumini* extracts had a MIC value of 0.05 µL/mL, which confirms their ability to inhibit fungal growth.

### 3.6 Antibacterial Activity

#### 3.6.1 Disc‐Diffusion Method

**FIGURE 4:**
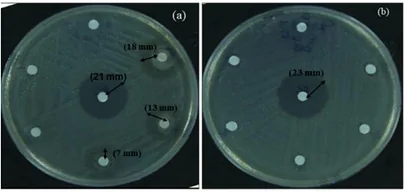
Antibacterial activity of methanolic extract of *S. cumini*: (a) pulp extract, (b) seed extracts.

The results of the antibacterial activity are shown in Figure 4. The methanol extract of *S. cumini* pulp exhibited maximum zone inhibition of 18 mm at a 100% dose, and seeds did not show a zone of inhibition (0 mm up to 100 % dose) against *E. coli* as compared to the positive control (maximum zone of inhibition average 24 mm at 10 µg doses). From the results, it was interpreted that the methanolic extract of *S. cumini* pulp could be a potent antibacterial agent.

#### 3.6.2 MIC Determination

The antibacterial activity of *S. cumini* seeds and pulp extract was evaluated by determining the MIC against *E. coli*. The MIC value for the pulp extract of *S. cumini* extract was found to be 180 µL/mL, which confirms its ability to inhibit bacterial growth. The seed extracts did not show any inhibition zone during the agar diffusion assay, suggesting low or negligible antibacterial activity.

### 3.7 Antiacne Activity

#### 3.7.1 Disc Diffusion Method

**FIGURE 5:**
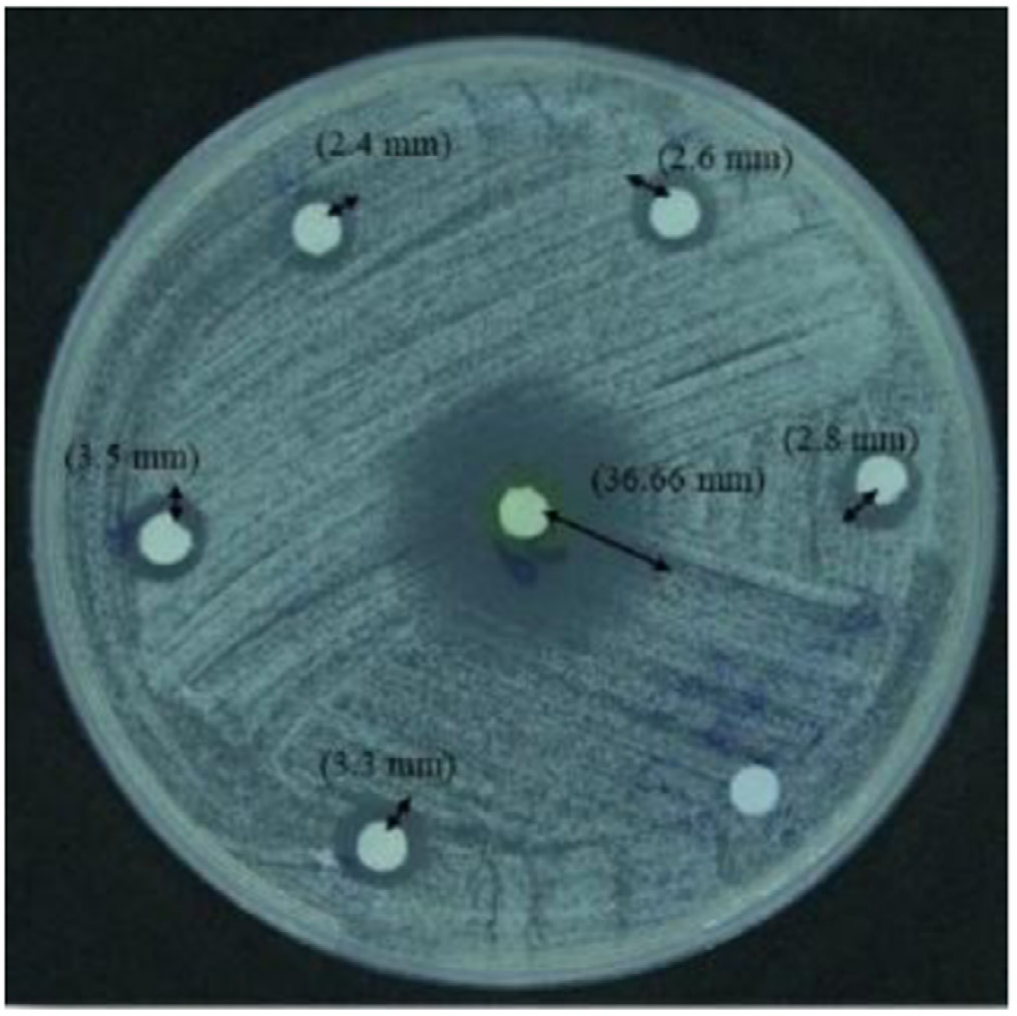
Antiacne activity of methanolic extract of *S. cumini* for pulp extract.

In this study, the disc diffusion method was used to assess the antiacne activity of *S. cumini* pulp against *P. acnes*. *P. acnes* are comparatively slow‐growing, aerotolerant, anaerobic Gram‐positive bacterium (rod form) connected with acne skin disease. Figure 5 depicts the inhibitory zones of each disc in the presence of various percentages of concentrations (0%, 6.25%, 12.5%, 25%, 50%, and 100% dose) of pulp. Based on the results obtained from this study, it was found that the sample of *S. cumini* pulp showed the maximum zone of inhibition (3.5 mm at 100 % dose) active against the test organism *P. acnes*, as compared to the positive control (maximum zone of inhibition 36 mm at 10 µg dose). It was concluded that *S. cumini* pulp exhibited antiacne activity [37].

#### 3.7.2 MIC Determination

The antiacne activity of *S. cumini* seeds and pulp extract was evaluated by determining the MIC against *P. acnes*. The pulp extract of *S. cumini* showed an MIC value of 1000 µg/mL, while the seed extract demonstrated a lower MIC value of 500 µg/mL.

### 3.8 In Vitro Biological Assays (Antidiabetic Activity)

#### 3.8.1 *α*‐Amylase Inhibitory Assay

**FIGURE 6:**
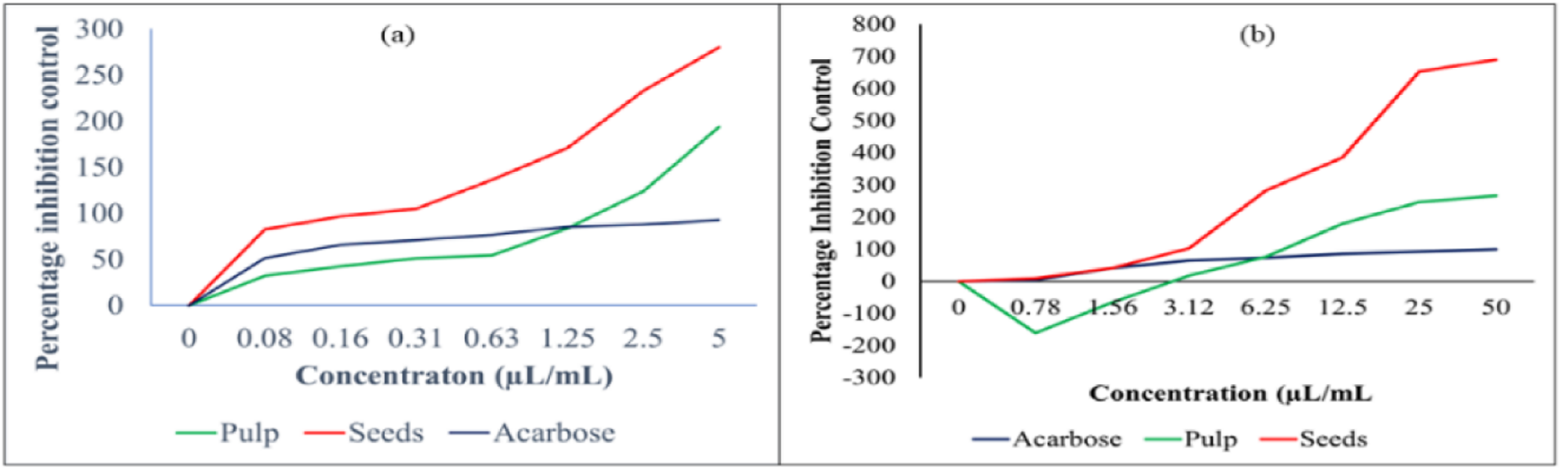
Inhibitory assay of both sample extract of *S. cumini*: (a) *α*‐Amylase; (b) *α*‐Glucosidase.

Antidiabetic activity of methanol extracts of both pulp and seeds of *S. cumini* was investigated to confirm their potential for usage in managing DM (diabetes mellitus). *α*‐Amylase, predominantly present in the saliva and pancreatic juice, is recognized as a vital enzyme in the digestive process. Targeting and inhibiting this enzyme is a viable solution to prevent high postprandial blood glucose levels. The methanolic extract of both pulp and seeds exhibited significant *α*‐amylase inhibitory activity, as shown in Figure 6a and Table S2. The seed extract revealed better activity as compared to the pulp extract. The highest level of inhibition was observed for the methanolic extract of pulp with an IC_50_ value of 0.238 µL/mL, while the seed extract showed an IC_50_ value of 0.05 µL/mL. Acarbose demonstrated an IC_50_ value of 0.60 µL/mL when employed as a positive control [38].

#### 3.8.2 *α*‐Glucosidase Inhibitory Assay

The *α*‐glucosidase enzyme is one of the essential enzymes of digestion, located in the mucosal brush border of the small intestine. Its inhibition is an effective solution to delay glucose absorption and prevent high postprandial blood glucose levels, which may probably suppress diabetes progression. The inhibitory activity of methanolic extracts of pulp and seeds of *S. cumini* on the *α*‐glucosidase enzyme was investigated, and the results are shown in Figure 6b and Table S3. The inhibition of the α‐glucosidase enzyme appears to be dose‐related, as the concentration of both extracts clearly affects the amount of enzyme inhibited. The calculated IC_50_ values for pulp and seed extracts are 4.80 µL/mL and 1.65 µL/mL, respectively, which indicates that the inhibitory potential of seed methanolic extract is better than that of methanolic extract pulp. Acarbose was used as a positive control, which showed an IC_50_ value of 2.50 µL/mL.

#### 3.8.3 ROS (Reactive Oxygen Species) Measurement

**FIGURE 7:**
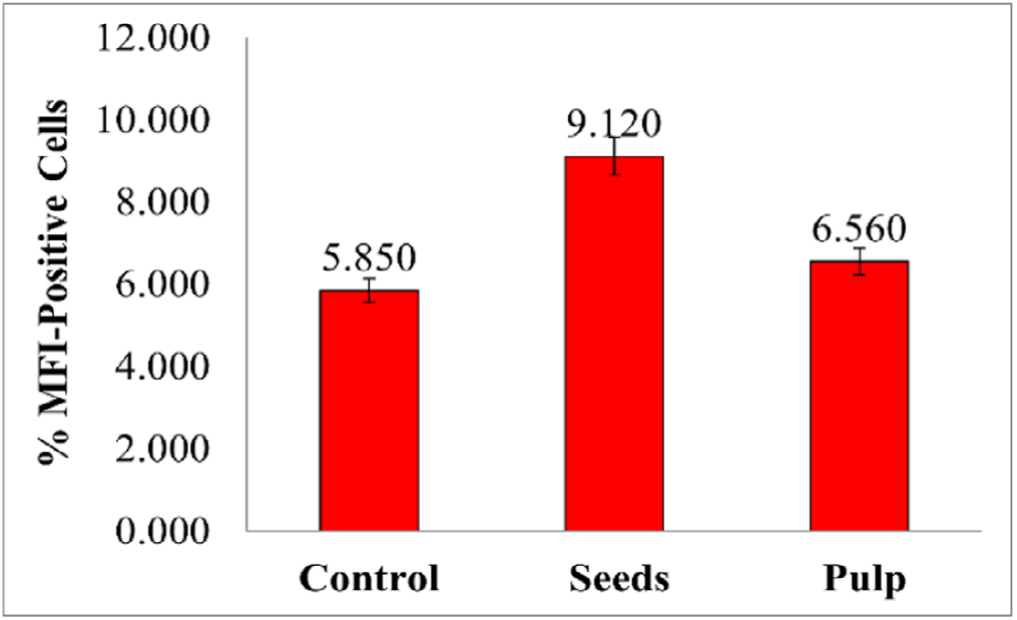
ROS measurement of methanolic extract of *S. cumini* pulp and seed extracts.

The results of ROS measurement in the L6 cell line and the MFI‐positive cells exposed to both pulp and seeds of the methanolic extract of *S. cumini* are shown in Figure 7 and Table S4. The ROS measurement was found to increase in a dose‐dependent manner. The highest ROS production was observed in the methanol extract of seeds (9.12%) as compared to pulp (6.56%).

#### 3.8.4 Insulin Secretion Assay

**FIGURE 8:**
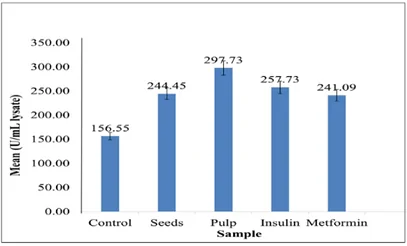
Insulin secretion assay of *S. cumini* pulp and seed extracts.

Insulin plays a crucial role in controlling blood glucose concentration. It promotes glucose uptake into cells by facilitating the translocation of glucose transporters (GLUT4) to the cell membrane. The results of the insulin secretion assay conducted on the extracts of *S. cumini* seeds and pulp are shown in Figure 8 and Table S5. According to the data obtained, the quantitative estimation of insulin showing highest insulin secretion level in the cell lysate treated with *S. cumini* pulp extract (297.73 U/mL Lysate), with respect to control cells without treatment (156.55 U/mL cell lysate) and positive control insulin (257.73 U/mL Lysate), in comparison to *S. cumini* seed extract. This indicates that *S. cumini* pulp extract has more pronounced insulin secretion than the seed extract.

#### 3.8.5 Glucose Uptake Assay

**FIGURE 9:**
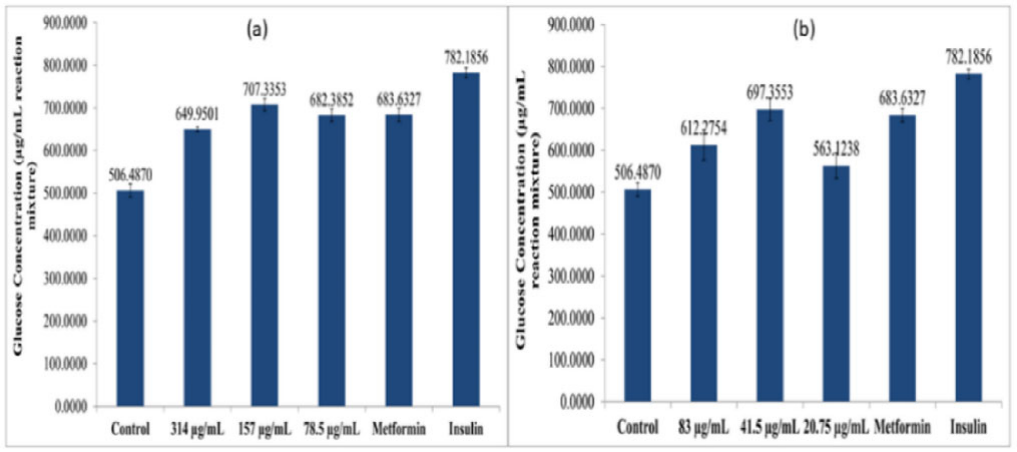
In Vitro glucose uptake activity of *S. cumini*: (a) Pulp extract (b) seed extract.

The results of the in vitro glucose uptake assay of both the methanol extracts of *S. cumini* on the L6 cell line are shown in Figure 9 and Table S6. The results were compared with metformin and insulin, which were used as standard antidiabetic drugs. When cells were treated with different concentrations of the extracts, then glucose concentration was measured along with control (cells without treatment) and standard insulin in the cell lysate. The results showed that the glucose concentration in cell lysate treated with *S. cumini* seeds extract at 41.5 µg/mL was found to be 697.35 µg/mL with respect to the control (506.48 µg/mL) and positive control‐insulin (782.18 µg/ml), showing a positive effect of the sample. On the other hand, *S. cumini* pulp extract at 157 µg/mL showed a glucose concentration of 707 µg/mL with respect to the control (506.48 µg/mL) and positive control‐insulin (782.18 µg/mL). The results suggest that the *S. cumini* pulp extract is more prominent than the seed extract.

#### 3.8.6 Cytotoxicity Assay

**FIGURE 10:**
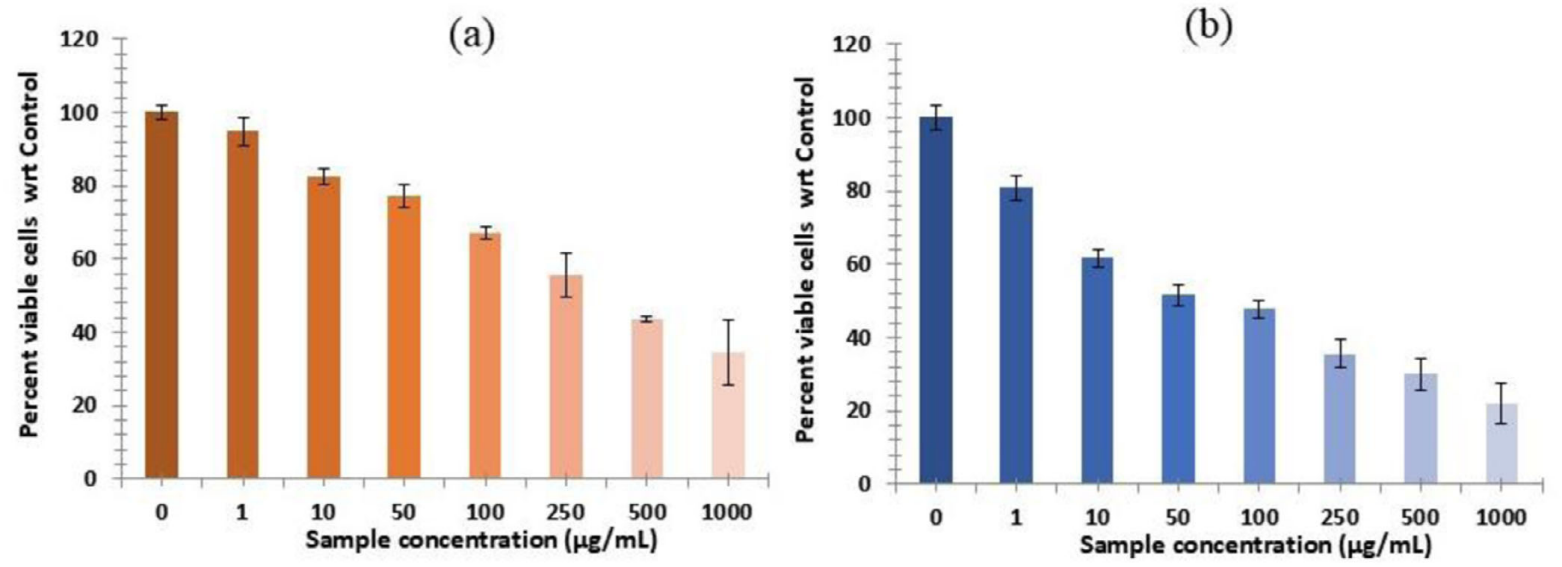
Cytotoxicity assay of *S. cumini*: (a) pulp extract (b) seed extracts.

The anticytotoxic effect of methanol extracts of both pulp and seeds of *S. cumini* against the L6 cell line (myoblast cell line) is represented in Figure 10a,b and Table S7. At varying concentrations; there were notable differences in the proportion of viable cells in both the pulp and seed extracts. On L6 cell line, the pulp extract showed 34.48% viability at 1000 µg/mL, but the seed extract showed 22.14 % viability. However, at a lower concentration of 1 µg/mL, the pulp extract showed 94.82 % vitality, while the seed extract showed 80.82% viability. The decline in cell viability was observed in a concentration‐dependent manner. The inhibitory concentration (IC_50_) values obtained by the MTT assay for pulp and seeds were 314 ± 0.09 µg/mL and 83.9 ± 0.1 µg/mL, respectively. Interestingly, both pulp and seed extracts did not show any toxic effects at lower concentrations, and less cytotoxicity was found in the increased concentration of pulp and seed extracts. Hence, it can be inferred from the results that at a limited dosage, both the pulp and seeds are biocompatible and are not harmful to human beings as well as other living organisms [39].

## 4 Conclusion

The present investigation of the wild ecotype of *S. cumini* extracts revealed the presence of a wide range of bioactive secondary metabolites (phenolic compounds, flavonoids, lignans, terpenoids, and alkaloids) as confirmed by GC‐MS and UHPLC‐QTOF‐MS. Both the extracts exhibited antidiabetic, antioxidant, and antibacterial activities. Cytotoxicity assay clearly showed the non‐toxic/biocompatible potential of *S. cumini* pulp and seeds. Future studies should focus on in vivo testing, detailed toxicological evaluations, and exploration of the fruit's broader therapeutic potential. Overall findings suggest that the wild ecotype fruits of *S. cumini*, which are not consumed by humans and animals, may have the potential to develop this eco variety in the Indian system of medicine.

## Author Contributions

**Ritu Sharma**: writing, conceptualization, review, editing, data analysis. **Jai Krishna Singh**: experimental work, writing draft. **Umesh Pal**: experimental work, writing draft. **Meenakshi Tanwar**: review, editing. **Rajinder K. Gupta**: conceptualization, supervision, review, and editing.

## Funding

The authors have nothing to report.

## Ethics Statement

This article does not contain any studies with human or animal participants. Ethical approval was not required.

## Conflicts of Interest

The authors declare no conflicts of interest.

## Supporting information



The Tables S1–S7 are available in the Supporting Information.
**Supporting File 1**: cbdv71627‐sup‐0001‐SuppMat.docx.

## Data Availability

The data that support the findings of this study are available on request from the corresponding author. The data are not publicly available due to privacy or ethical restrictions.

## References

[1] H. Singh , V. Sharma , B. Sharma , M. Kumar , and N. Rana , “Jamun (*Syzygium cumini* L.) Seeds: A Review on Phytochemistry, Pharmacology, Nutritional Profile and Traditional Uses,” YMER 5, no. 5 (2020): 547–559.

[2] G. C. Jagetia , “Bioactive Phytoconstituents and Medicinal Properties of Jamun (*Syzygium cumini*),” Journal of Exploratory Research in Pharmacology 9, no. 3 (2024): 180–212.

[3] A. H. Sciences , “A Comprehensive Review of the Effects of *Psidium Guajava* and *Syzygium cumini* Leaves on Diabetes Mellitus,” Preventive Nutrition and Food Science 30, no. 06 (2025): 209–221. 40612758 10.3746/pnf.2025.30.3.209 PMC12213247

[4] J. Anon (*Syzygium cumini*), (https://agritech.tnau.ac.in/horticulture/horti_fruits_jamun.html), (2008).

[5] C. I. Chukwuma , M. S. Islam , and E. O. Amonsou , “A Comparative Study on the Physicochemical, Antioxidative, Antihyperglycemic and Antilipidemic Properties of Amadumbe (*Colocasia esculenta*) and Okra (*Abelmoschus esculentus*) Mucilage,” Journal of Food Biochemistry 42, no. 5 (2018): 1–12.

[6] K. Prabakaran and G. Shanmugave , “Antidiabetic Activity and Phytochemical Constituents of *Syzygium Cumini* Seeds in Puducherry Region, South India,” International Journal of Pharmacognosy Phytochemical Research 9, no. 07 (2018): 7–11.

[7] T. Beniwal , R. Sharma , and R. K. Gupta , “Phytochemical, Antioxidant and Antimicrobial Activity of *Livistona Chinensis* (Jacq.) R.Br. ex Mart. Seeds,” Indian Journal of Traditional Knowledge 23, no. 2 (2024): 162–169.

[8] R. Sharma , D. Kumar , and R. K. Gupta , “Bioactive Profiling of Two Varieties of Indian Legumes: Adzuki and Mung Beans,” International Journal of Food Science and Technology 59, no. 9 (2024): 6218–6230.

[9] J. Parekh and S. Chanda , “Antibacterial and Phytochemical Studies on Twelve Species of Indian Medicinal Plants,” African J Biomed Res 10, no. 2 (2010): 175–181.

[10] J. C. Christenson and R. F. R. Korgenski , Laboratory Diagnosis of Infection Due to Bacteria, Fungi, Parasites, and Rickettsiae, ed. Sarah S. Long and Charles G. Prober MF (Elsevier, 2018).

[11] P. W. Fowler , C. Wright , H. Spiers , et al., “A Crowd of BashTheBug Volunteers Reproducibly and Accurately Measure the Minimum Inhibitory Concentrations of 13 Antitubercular Drugs From Photographs of 96‐Well Broth Microdilution Plates,” eLife 11 (2022): e75046. 35588296 10.7554/eLife.75046 PMC9286738

[12] S. Sathiyaraj , G. Suriyakala , A. Dhanesh Gandhi , et al., “Biosynthesis, Characterization, and Antibacterial Activity of Gold Nanoparticles,” Journal of Infection Public Health 14, no. 12 (2021): 1842–1847. 34690096 10.1016/j.jiph.2021.10.007

[13] J. Hudzicki , “Kirby‐Bauer Disk Diffusion Susceptibility Test Protocol Author Information,” American Society for Microbiology 15, no. 1 (2009): 1–23.

[14] M. Telagari and K. Hullatti , “In Vitro *α*‐amylase and *α*‐Glucosidase Inhibitory Activity of *Adiantum Caudatum* Linn. And *Celosia Argentea* Linn. Extracts and Fractions,” Indian Journal of Pharmacology 47, no. 4 (2015): 425–429. 26288477 10.4103/0253-7613.161270 PMC4527066

[15] M. M. Al‐Oqail , “Anticancer Efficacies of *Krameria Lappacea* Extracts Against Human Breast Cancer Cell Line (MCF‐7): Role of Oxidative Stress and ROS Generation,” Saudi Pharmaceutical Journal 29, no. 3 (2021): 244–251. 33981173 10.1016/j.jsps.2021.01.008 PMC8084728

[16] N. Kaur , L. Kishore , and R. Singh , “ *Dillenia Indica* L. Attenuates Diabetic Nephropathy via Inhibition of Advanced Glycation End Products Accumulation in STZ‐Nicotinamide Induced Diabetic Rats,” Journal of Traditional Complement Medicines 8, no. 1 (2018): 226–238. 10.1016/j.jtcme.2017.06.004 PMC5756019 29322013

[17] D. Vishwanath , H. Srinivasan , M. S. Patil , et al., “Novel Method to Differentiate 3T3 L1 Cells In Vitro to Produce Highly Sensitive Adipocytes for a GLUT4 Mediated Glucose Uptake Using Fluorescent Glucose Analog,” Journal of Cell Communication and Signaling 7, no. 2 (2013): 129–140. 23292944 10.1007/s12079-012-0188-9 PMC3660688

[18] V. J. Meerloo , G. J. Kaspers , and J. Cloos , “Cell Sensitivity Assays: The MTT Assay,” Methods in Molecular Biology 731 (2011): 237–245. 21516412 10.1007/978-1-61779-080-5_20

[19] P. Karak , “Biological Activities of Flavonoids: An Overview,” International Journal of Pharmaceutical Sciences and Research 10, no. 4 (2019): 1567–1574.

[20] M. Fraga‐Corral , P. Otero , J. Echave , et al., “By‐Products of Agri‐Food Industry as Tannin‐Rich Sources: A Review of Tannins' Biological Activities and Their Potential for Valorization,” Foods 10, no. 1 (2021): 1–22. 10.3390/foods10010137 PMC7827785 33440730

[21] S. D. Desai , D. G. Desai , and H. Kaur , “Saponins and Their Biological Activities,” Pharma Times 41, no. 3 (2009): 13–16.

[22] A. Gupta , E. Jeyakumar , and R. Lawrence , “Pyrogallol: A Competent Therapeutic Agent of the Future,” Asian Journal of Microbiology, Biotechnology & Environmental Sciences 23, no. 2 (2021): 213–217.

[23] T. Sudha , S. Chidambarampillai , and V. R. Mohan , “GC‐MS Analysis of Bioactive Components of Aerial Parts of *Fluggea Leucopyrus* Willd. (Euphorbiaceae),” Journal of Applied Pharmaceutical Science 3, no. 05 (2013): 126–130.

[24] M. El Ghozlani , L. Bouissane , M. Berkani , et al., “Synthesis and Biological Evaluation Against *Leishmania donovani* of Novel Hybrid Molecules Containing Indazole‐Based 2‐Pyrone Scaffolds,” MedChemComm 10, no. 1 (2019): 120–127. 30774860 10.1039/c8md00475g PMC6350763

[25] M. Azizian , S. Gheshlaghi , A. Danesh , F. Forouzanfar , and A. Shakeri , “α‐Pyrones: Natural Occurrence, Chemistry, and Biological Approaches‐ An Update,” Revista Brasileira de Farmacognosia 34, no. 6 (2024): 1201–1217.

[26] L. T. Marton , L. M. Pescinini‐e‐Salzedas , M. E. Camargo , et al., “The Effects of Curcumin on Diabetes Mellitus: A Systematic Review,” Frontier Endocrinology 12 (2021): 1–12. 10.3389/fendo.2021.669448 PMC8126655 34012421

[27] B. Jyotirmayee and G. Mahalik , “A Review on Selected Pharmacological Activities of *Curcuma Longa* L,” International Journal of Food Properties 25 (2022): 1377–1398.

[28] C. Augustine , “Unravelling the Competence of Leucocyanidin in Free Radical Scavenging: A Theoretical Approach Based on Electronic Structure Calculations,” Journal of Structural Chemistry 60 (2019): 198–209.

[29] A. Saifi , R. Chauhan , and J. Dwivedi , “An Exploratory Study on *Ficus Bengalensis* Linn,” Research Journal of Pharmacognosy and Phytochemistry 6, no. 3 (2014): 1–7.

[30] W. Chen and A. M. Viljoen , “Geraniol—A Review of a Commercially Important Fragrance Material,” South African Journal of Botany 76, no. 4 (2010): 643–651.

[31] X. Wang , Y. Jia , and H. He , “The Role of Linoleic Acid in Skin and Hair Health: A Review,” International Journal of Molecular Sciences 26 (2025): 246. 10.3390/ijms26010246 PMC11719646 39796110

[32] F. Rouvier , L. Abou , E. Wafo , and J. M. Brunel , “Linoleic Fatty Acid from Rwandan Propolis: A Potential Antimicrobial Agent Against *Cutibacterium Acnes* ,” Current Issues in Molecular Biology 47 (2025): 162. 40136416 10.3390/cimb47030162 PMC11941583

[33] S. P. Jaramillo , J. Calva , A. Jiménez , and C. Armijos , “Isolation of Geranyl Acetate and Chemical Analysis of the Essential Oil From *Melaleuca armill*aris (Sol. ex Gaertn.) Sm,” Applied Sciences 14 (2024): 1864.

[34] I. Krasteva , M. Yotova , D. Yosifov , N. Benbassat , K. Jenett‑Siems , and S. Konstantinov , “Cytotoxicity of Gypsogenic Acid Isolated From *Gypsophila Trichotoma* ,” Pharmacognosy Magazine 10, no. 38 (2014): S430–S433. 24991123 10.4103/0973-1296.133299 PMC4078331

[35] R. Upadhyay and C. Singh , Polyphenol Nutraceuticals and Healthcare, (1st ed.) (Eds.). (CRC Press, 2026), 10.1201/978100358850.

[36] H. P. Gajera , S. N. Gevariya , D. G. Hirpara , S. V. Patel , and B. A. Golakiya , “Antidiabetic and Antioxidant Functionality Associated With Phenolic Constituents From Fruit Parts of Indigenous Black Jamun (*Syzygium cumini* L.) Landraces,” Journal of Food Science and Technology 54, no. 10 (2017): 3180–3191. 28974803 10.1007/s13197-017-2756-8 PMC5602981

[37] S. S. Joo , J. S. K. ang , S. G. Kim , J. Choi , and K. W. Hwang , “Antiacne Activity of *Selaginella Involvens* Extract and Its Non‐Antibiotic Antimicrobial Potential on *Propionibacterium Acnes* ,” Phytotherapy Research 339, no. 10 (2008): 335–339. 10.1002/ptr.2319 17926337

[38] R. R. Franco , L. F. Ribeiro Zabisky , J. Pires de Lima Júnior , et al., “Antidiabetic Effects of *Syzygium cumini* Leaves: A Non‐Hemolytic Plant With Potential Against Process of Oxidation, Glycation, Inflammation and Digestive Enzymes Catalysis,” Journal of Ethnopharmacology 261, no. 05 (2020): 113132. 32673709 10.1016/j.jep.2020.113132

[39] G. Fotakis and J. A. Timbrell , “In Vitro Cytotoxicity Assays: Comparison of LDH, Neutral Red, MTT and Protein Assay in Hepatoma Cell Lines Following Exposure to Cadmium Chloride,” Toxicology Letters 160, no. 2 (2006): 171–177. 16111842 10.1016/j.toxlet.2005.07.001

