# Supplementary material for: Ethnopharmacological Evaluation of a Wild Ecotype of Syzygium cumini (L.) Skeels (Jamoya) Fruit
Source: Chem Biodivers. 2026 Aug 25;23(8):e71627. doi: 10.1002/cbdv.71627 (PMC13506090; doi:10.1002/cbdv.71627)
Supplement: Supplementary file 1 [file CBDV-23-e71627-s001.docx]

**Supplementary File**

1. GC-MS analysis

GC-MS (Shimadzu, GCMS-QP2010) was used to determine the volatile components in crude methanol extract from *S. cumini* seeds and pulp. Helium was used as a carrier gas in split mode at 260 °C when the sample was injected into the GC-MS. While electron ionization (EI) was used as the ionization method, the column flow rate was sustained at 1.21 mL/min. Temperatures at contact and the ion source were 220 ^o^ C and 270 ^o^ C respectively. The oven temperature was set from 80°C for 2 minutes to 300 °C for 21 minutes with a solvent delay time of 3.50 minutes. NIST (National Institute of Standards and Technology) mass spectrum data was utilized to locate the separated peaks.

2. UHPLC-QTOF-MS analyses

The bioactive metabolites of *S. cumini* were analyzed using the UHPLC-QTOF-MS by following the protocol previously described by Perumal et al. The mass spectroscopic analysis was carried out only in positive mode (ESI+). The UHPLC system was assembled with an AD autosampler, and the column used was a Waters nano Acquity HSS paired with a quadrupole time-of-flight mass spectrometer (QTOF-MS). An Accucore C18 column measuring 100 mm by 3 mm made up the analytical column. Subsequently, a binary mobile phase comprising various components labeled as A (water containing 0.1% formic acid) and B (1% formic acid with acetonitrile) was used to elute 5 μL of methanol extracts (1 mg/mL) into the column. A linear gradient with a rate of 0.2 mL/min has been determined for a 25 min program. In these conditions, the scanning time was roughly 1 second. The components in both samples were identified using ChemSpider software.

3. Antioxidant Activity

The free radical scavenging activity of the extract was evaluated by following the previously reported method, with some modifications. Antioxidant activity was described using the 1,1-diphenyl-2-picrylhydrazyl (DPPH) method. 5 µL methanol extract of different concentrations (0 µg/mL, 0.08 µg/mL, 0.16 µg/mL, 0.31 µg/mL, 0.63 µg/mL, 1.25 µg/mL, 2.5 µg/mL and 5 µg/mL) were mixed with 0.1 mL of a 0.1 mM DPPH solution in 96-well plates. After 30 minutes of incubation in the dark, the absorbance of each sample was measured at 495 nm. The control was a reaction mixture containing 20 μL of deionized water.

4. Antifungal activity

The antifungal activity of *S. cumini* seeds and pulp methanolic extracts was evaluated against *Candida albicans (C. albicans)*, following the method described by Sathiyaraj et al. Sabouraud dextrose agar (SDA) petri plates were used for fungal cultures. SDA plates were inoculated by spreading 100 µL of fungal culture (obtained from Microbial Type Culture Collection (MTCC), Chandigarh, India). Wells were made in each SDA plate and then placed with 10 µL of different concentrations (0%, 6.25%, 12.5%, 25%, 50%, and 100%). 50 µg of amphotericin B was used as the positive control. At 37 °C, the fungal plates were incubated for 24 hours. The zone of inhibition was determined after the incubation period.

Anti-acne activity assay

5. Antibacterial activity

The antibacterial activity of the methanol extract of *S. cumini* was done using the disc diffusion method. The MHA (Mueller Hinton agar) plates were inoculated with 100 µL of bacterial culture *Escherichia coli (E. coli)* (obtained from Microbial Type Culture Collection (MTCC), Chandigarh, India, adjusted to 0.5 McFarland Unit - approximate cell density (1.5 × 108 CFU/mL) and then placed discs containing 10 µL of varied concentration (0- 100 mg/mL). 10% of the sample was obtained and serially diluted to achieve the required volume to load onto the disc. One disc in each plate was loaded with solvent alone as vehicle control, while a Ciprofloxacin disc (10 µg) was used as a positive control. The *E. coli* plates were incubated at 37 °C for 24 hours. After the incubation period, the zone of inhibition was measured.

6. Anti-acne activity

The disc diffusion method is used to determine the anti-acne activity in the methanolic extract of *S. cumini*. The MHA petri plates were used. 100 µL of bacterial culture (obtained from Microbial Type Culture Collection (MTCC), Chandigarh, India) *Propionibacterium acnes (P. acnes)*, was kept in sterilized MHA petri plates. Wells of identical size were prepared in agar plates, and then discs were placed with 10 µL of various concentrations (0 %, 6.25 %, 12.5 %, 25 %, 50 %, and 100 %). 10 µg of Ciprofloxacin was employed as the positive control. The petri plates were incubated for 24 hours at 37 °C. The zone of inhibition was determined after the incubation period.

7. Antidiabetic activities

α-Amylase inhibition assay

The previously reported α-amylase inhibition determination method was used with some modifications. The 96-well microplate was used to conduct the amylase activity. A pH of 6.9 was maintained by forming a sodium phosphate buffer with 0.6 M NaCl. A 96-well microplate was filled with each well containing 20 μL of soluble starch (1% w/v, 20 mg/mL), 10 μL of plant extract (1 mg/mL), and 20 μL of acarbose. The mixture was then incubated at 37 ^0^C for 10 min. Following incubation, in a reaction mixture, 10 μL of α-amylase (12 mg/1 mL) was added, and it was then incubated for a further 15 minutes at 37 ^0^C. 100 μL of iodine reagent and 10 μL of HCl (1M) were added. Two distinct wells were utilized for the negative and positive controls: 20 μL methanol and 50 μg/mL acarbose. Using a microplate reader, absorbance was determined at 620 nm.

8. α-Glucosidase inhibitory activity

The α-glucosidase inhibition assay was carried out by Rauf et al. (2021) with some modifications. A phosphate buffer solution of 100 mM with a pH of 6.8 was prepared. A reaction mixture comprising 50 μL of phosphate buffer (100 mM, pH = 6. 8), 10 mL of α-glucosidase (1 U/mL), and 20 μL of extract at different concentrations (0-1000 μg/mL) was pre-incubated for 15 min. at 37 °C in a 96-well plate. Then, a substrate of 20 μL P-NPG (5 mM) was added, and the mixture was further incubated for 20 minutes at 7 °C. To stop the reaction, 50 μL of 0.1 M Na_2_CO_3_ was added. The ELISA microplate reader will be used to measure the absorbance of the released p-nitrophenol at 405 nm. Acarbose was utilized as a positive control at 1 mg/mL.

9. Reactive oxygen species activity (ROS) measurement

To produce ROS, 2'-7'-Dichlorodihydrofluorescein diacetate (DCFH-DA) probe was used to produce ROS. L6 cells were cultured for 24 hrs at 37 °C after being plated in 6 well plates at a density of 5000–8000 cells/well in 1 mL of Dulbecco’s Modified Eagle Medium (DMEM) with 1% antibiotic solution and 10% FBS (fetal bovine serum). Then cells were incubated for a further 24 hrs at varying concentrations. The medium was withdrawn after incubation, and cells were collected using trypsin-EDTA in a 1.5 mL tube, and then once again washed with 500 μL of cold PBS. Further, a cell pellet was added to 100 μL of PBS (Phosphate Buffered Saline) containing 2 μM DCFDA, and the samples were collected in an hour using a BD FACS Calibur flow cytometer. The acquired data were examined using the 2.5.1 version of Flowing.

10. Insulin secretion assay

Insulin secretion in cells was determined by following the previously described method (using Dia Metra Insulin Elisa- Cat No.- DKO076 kit) with slight modifications. For this purpose, the cells were lysed in 50 µL buffer in each well of a 96-well plate, 200-500 µL for 1 well of a well plate or 50 mM dish plate. After that, it was centrifuged for 5 minutes at 10,000 rpm, and the supernatant was collected. Then, 100 µL metformin standard sample, 100 µL extract sample (both pulp and seeds), and 100 µL detection antibody (conjugated detection antibody) solution was added to each well, and then the plate was sealed and incubated for 2 hrs at room temperature. After that, the plate was washed with wash buffer four times, and the buffer was blotted by firmly tapping the plate upside down on absorbent paper. 100 µL of 3,3’,5,5’-tetramethylbenzidine (TMB) solution was added and incubated for 15 minutes in the dark. To stop the reaction, 100 µL of stop solution was added to each well, and the absorbance was measured at 450 nm within 5 minutes.

11. Glucose uptake assay

The glucose uptake of extracts from *Syzygium cumini* seeds and pulp was determined using a slightly modified technique mentioned by Gudise et al. The L6- cells (L6- Rat skeletal muscle myoblast cell line, RRID- CVCL_0477, obtained from NCCS, Pune, India) were plated in 96-well cell culture plates at a density of 8 × 104 cells/well and incubated for 24 hrs in a standard culture medium. Cells were handled as positive control and then cultured in glucose-free DMEM (Dulbecco's Modified Eagle Medium-AT149-1L) for one hour after being twice washed with Krebs-Ringer-phosphate-HEPES (KRPH) buffer (20 mM HEPES, 5 mM KH_2_PO_4_, 1 mM CaCl_2_, 136 mM NaCl and 4.7 mM KCl). The cells were then treated for 40 minutes in the presence or absence of 10 mM 2-DG for 20 minutes in a KRPH buffer containing 2% (v/v) bovine serum albumin. Following three PBS washes to eliminate exogenous 2-DG, cells were lysed using extraction buffer, centrifuged at 500 rpm for two minutes, and heated to 85°C for 40 minutes to destroy endogenous nicotinamide adenine dinucleotide phosphate (NADP+). The 2-deoxyglucose-6-phosphate (2-DG6P) concentration of the resultant supernatant was determined using the GOD-POD Enzyme Assay Kit, and the plate was read at 505 nm using a microplate reader. To get the blank value, lysates of cells that had not been treated with 2-deoxyglucose were examined. By comparing the data to a standard, nanomoles of 2-deoxy glucose were estimated. The standard was 2-DG6P, authentic. 0.1 U/mL of insulin was employed as the positive control and 1 mg of metformin as the negative control.

12. Cytotoxicity assay

The cytotoxicity of fruit extracts on L6 cell line (L6- Rat skeletal muscle myoblast cell line, RRID- CVCL_0477, obtained from NCCS, Pune, India) was determined by following the procedure described by Kim et al., with few modifications. The cells (10000 cells/well) were cultured on a 96-well plate in DMEM (Dulbecco's Modified Eagle Medium) containing 1% antibiotic solution, 10% FBS (fetal bovine serum) and 37 °C in an incubator with 5% CO_2_ for a whole day. In addition, various sample concentrations (0µg/mL, 1µg/mL, 10 µg/mL, 50 µg/mL, 100 µg/mL, 250 µg/mL, 500 µg/mL and 1000 µg/mL) were added to the cells. After 24 hrs, 10 µL of MTT solution was placed in each well and incubated for an extra 2 hours. Then, 100 µL of dimethyl sulfoxide (DMSO) was added for the removal of the culture supernatant and the dissolution of the cell layer matrix. The sample was then measured at 540 nm and 660 nm using an Elisa plate reader.

13. Antioxidant activity (DPPH radical scavenging activity)

| Table S1- DPPH Scavenging activity of methanolic extract of *S. cumini* pulp and seeds extract | | |
| --- | --- | --- |
| **Sample Concentration**  **(µL/mL)** | **%Inhibition** | |
|  | **Jamun pulp extract** | **Jamun seeds extract** |
| 0.78125 | 11.12455 ± 4.58 | 23.20113 ± 1.73 |
| 1.5625 | 18.77267 ± 1.53 | 55.72238 ± 2.14 |
| 3.125 | 69.98186 ± 7.02 | 75.15581 ± 0.66 |
| 6.25 | 87.87787 ± 0.61 | 79.77337 ± 1.17 |
| 12.5 | 89.51028 ± 0.36 | 84.81586 ± 0.85 |
| 25 | 88.45224 ± 0.85 | 87.84703 ± 5.05 |
| 50 | 93.8029 ± 0.94 | 100.5382 ± 4.42 |

14. α-Amylase inhibitory assay

| **Table S2:** α-Amylase inhibitory assay of pulp and seeds extract of *S. cumini* | | | |
| --- | --- | --- | --- |
| **Sample Concentration**  **(µL/mL)** | **%Inhibition** | | |
|  | **Jamun pulp extract** | **Jamun seeds extract** | **Acarbose** |
| 0.08 | 32.4042 ± 5.62 | 82.5843 ± 6.95 | 51.6598 ± 4.35 |
| 0.16 | 42.8571 ± 1.43 | 96.6292 ± 1.35 | 66.1826 ± 6.22 |
| 0.31 | 51.5679 ± 6.37 | 105.056 ± 2.80 | 71.1618 ± 5.72 |
| 0.63 | 54.7038 ± 1.64 | 136.517 ± 7.19 | 76.971 ± 2.07 |
| 1.25 | 83.9721 ± 9.72 | 171.348 ± 4.96 | 85.2697 ± 3.79 |
| 2.5 | 124.042 ± 2.40 | 233.146 ± 4.38 | 87.7593 ± 3.27 |
| 5 | 194.077 ± 1.66 | 280.337 ± 5.22 | 92.9461 ± 1.79 |

15. α-Glucosidase inhibitory assay

| **Table S3:** α-Glucosidase inhibitory assay of pulp and seeds extract of *S. cumini* | | | |
| --- | --- | --- | --- |
| **Sample Concentration**  **(µL/mL)** | **%Inhibition** | | |
|  | **Jamun pulp extract** | **Jamun seeds extract** | **Acarbose** |
| 0.78125 | -161.333 ± 2.48 | 8.84615 ± 8.73 | 3.96226 ± 9.27 |
| 1.5625 | -66 ± 2.21 | 40.7692 ± 3.28 | 41.1321 ± 4.11 |
| 3.125 | 17.3333 ± 6.53 | 102.692 ± 4.59 | 65.0943 ± 5.05 |
| 6.25 | 76 ± 3.44 | 282.308 ± 7.03 | 73.3962 ± 5.62 |
| 12.5 | 179.333 ± 6.28 | 385.769 ± 4.87 | 85.6604 ± 2.89 |
| 25 | 246.667 ± 2.11 | 653.077 ± 1.12 | 92.4528 ± 6.19 |
| 50 | 266.667 ± 3.44 | 689.615 ± 1.66 | 99.6226 ± 7.01 |

16. ROS (Reactive Oxygen Species) measurement

| **Table S4-** ROS measurement of methanolic extract of *S. cumini* pulp and seeds extract | |
| --- | --- |
| **Sample** | **MFI-Positive Cells (%)** |
| Control | 5.850 ± 3.55 |
| Jamun pulp extract | 6.560 ± 5.12 |
| Jamun seeds extract | 9.120 ± 5.23 |

17. Insulin secretion assay

| **Table S5-** Insulin secretion assay of *S. cumini* pulp and seeds extract | |
| --- | --- |
| **Sample** | **Mean (U/mL Lysate)** |
| Control | 156.55 ± 1.47 |
| Jamun pulp extract | 297.73 ± 1.02 |
| Jamun seeds extract | 244.45 ± 1.23 |
| Insulin | 257.18 ± 1.68 |
| Metformin | 241.09 ± 1.55 |

18. Glucose uptake assay

| **Table S6:** α-Glucosidase inhibitory assay of pulp and seeds extract of *S. cumini* | | |
| --- | --- | --- |
| **Sample Concentration**  **(µg/mL)** | **Glucose Conc. (µg/mL reaction mixture)** | |
|  | **Jamun pulp extract** | **Jamun seeds extract** |
| Control | 506.4870 ± 2.55 | 506.4870 ± 3.82 |
| 83 | 649.9501 ± 3.12 | 612.2754 ± 3.26 |
| 41.5 | 707.3353 ± 4.10 | 697.3553 ± 2.95 |
| 20.75 | 682.3852 ± 2.11 | 563.1238 ± 5.32 |
| Metformin | 683.6327 ± 1.88 | 683.6327 ± 2.12 |
| Insulin | 782.1856 ± 1.72 | 782.1856 ± 1.10 |

19. Cytotoxicity assay

| **Table S7:** Cytotoxicity assay of *S. cumini* (a) pulp extract (b) seeds extract | | |
| --- | --- | --- |
| **Sample Concentration**  **(µg/mL)** | **Percent viable cells w.r.t Control** | |
|  | **Jamun pulp extract** | **Jamun seeds extract** |
| 0 | 100 ± 3.38 | 100 ± 4.93 |
| 1 | 94.8276 ± 7.44 | 80.8219 ± 6.95 |
| 10 | 82.4713 ± 4.63 | 61.6438 ± 4.83 |
| 50 | 77.2989 ± 6.32 | 51.5982 ± 5.52 |
| 100 | 67.2414 ± 3.57 | 47.7169 ± 4.85 |
| 250 | 55.7471 ± 11.88 | 35.6164 ± 7.41 |
| 500 | 43.3908 ± 1.44 | 29.9087 ± 4.21 |
| 1000 | 34.4828 ± 1.79 | 22.1461 ± 1.11 |
